# Response to Water Stress of Eight Novel and Widely Spread Citrus Rootstocks

**DOI:** 10.3390/plants14050773

**Published:** 2025-03-03

**Authors:** Giulia Modica, Fabio Arcidiacono, Ivana Puglisi, Andrea Baglieri, Stefano La Malfa, Alessandra Gentile, Vicent Arbona, Alberto Continella

**Affiliations:** 1 Department of Agriculture, Food and Environment (Di3A), University of Catania, Via Santa Sofia 100, 95123 Catania, Italy; giulia.modica@unict.it (G.M.); fabio.arcidiacono@studium.unict.it (F.A.); ipuglisi@unict.it (I.P.); abaglie@unict.it (A.B.); stefano.lamalfa@unict.it (S.L.M.); alessandra.gentile@unict.it (A.G.); 2 Plant Ecophysiology and Biotechnology Laboratory, Department of Agricultural and Environmental Sciences, Universitat Jaume I, 12071 Castellon de la Plana, Spain; arbona@uji.es

**Keywords:** abiotic stress, irrigation, gas exchanges, malondialdehyde, enzymatic activity, hormones

## Abstract

Drought is a problematic abiotic stress affecting citrus crops in the Mediterranean basin and the rootstock plays a fundamental role in adopting adaptive mechanisms in response to water deficit. The aim of this study is to evaluate the response of eight rootstocks under three treatments imposed: control (100% of reference evapotranspiration, Et_0_), 66% Et_0_ and 50% Et_0_. The rootstock genotypes studied were C35 citrange, Bitters, Carpenter and Furr which have been recently spread and so far, little investigated, while others have been widely used especially in the Mediterranean citrus industry, i.e., *Citrus macrophylla*, *C. volkameriana*, Swingle citrumelo and Carrizo citrange. Morphological analyses, leaf chlorophyll content determination, physiological measurement, proline accumulation, malondialdehyde determination and antioxidant enzyme activities were measured. The results exhibited that Bitters and Furr showed an increment in leaf area to reduce the effects of drought conditions. A decrement in gas exchanges and xylem water potential was noticed in Carrizo and C35 citrange at both water shortage treatments. Carrizo exhibited a significant increase in malondialdehyde at both stresses (90.3 and 103.3%, for 66 and 50% Et_0_). Bitters and Furr performed better than the other rootstocks with regard to enzymatic and hormonal assays. Specifically, Bitters showed a significant reduction in CAT (−68.6%), SOD (−82.5%) and APX (−36.7%). Furthermore, Bitters and Furr were closely related to morphological parameters, e.g., leaf area and root length, and physiological measurements. *C. volkameriana* showed a decrease in xylem water potential, while overall Carrizo and C35 citranges showed a susceptible response to water stress reducing morphological and physiological measurements.

## 1. Introduction

Historically, the sour orange (*Citrus aurantium* L.) was the most widely used rootstock in the Mediterranean region because of its high tolerance to various biotic and abiotic stresses. The citrus industry has been seriously threatened by the spread of the citrus tristeza virus (CTV) as sour oranges exhibited high sensitivity to this disease. So, new plantations were established using citranges [*Citrus sinensis* (L.) Osb. × *Poncirus trifoliata* (L.) Raf.] and other trifoliate orange hybrids, i.e., Swingle citrumelo, equally tolerant to CTV. Nevertheless, these rootstocks are sensitive to several environmental conditions and biotic stresses. For this reason, the study of new rootstocks under different abiotic conditions is important for many citrus-producing countries [1,2].

Water stress is a critical factor that significantly limits productivity worldwide in citrus [3,4]. Previous researchers have emphasized the pivotal role of several citrus rootstock genotypes with different tolerances to water scarcity and their different responses to irrigation management [5,6,7].

Some morphological and physiological measurements are indicators of the resilience that the rootstock may confer to the plant in relation to drought stress [8]. For example, stomatal closure is an important response caused by water stress to prevent excessive water loss [8]. Specifically, some studies reported that several citrus genotypes, e.g., Swingle citrumelo, do not activate physiological mechanisms to mitigate water stress, such as reducing the leaf area of the canopy to minimize water consumption under drought conditions [9]. Chlorophyll degradation is an important marker of stress, and the SPAD index is widely used to identify plant stress symptoms that alter the photosynthetic process, e.g., reported in lemon and orange [10,11,12,13]. Chlorophyll decay may be attributed to oxidative damage caused by an excess of reactive oxygen species (ROS) generated by abiotic stress [13,14]. Citrus species are highly sensitive to prolonged drought, which induces significant physiological and metabolic imbalances [8,15,16]. Nevertheless, some citrus rootstocks, such as Rangpur lime and Swingle citrumelo, exhibit adaptive mechanisms to counteract drought, allowing the plant to maintain tissue water potential close to unstressed levels [17,18]. Hormones are also involved in the response of plants to abiotic stress, such as in the case of abscisic acid (ABA) which takes part in regulating citrus responses to water stress. Specifically, ABA plays an important role in limiting damage due to water deficit in plants [19,20,21]. Indeed, previous researchers noticed that jasmonic acid (JA) performs a dual role, being involved both in the regulation of leaf senescence and in plant adaptive responses to water deficiency [22,23,24].

In this context, the aim of this study is to investigate the effect of two levels of water stress compared to the full irrigation on eight rootstock genotypes, among which some were recently released and poorly investigated, evaluating their physiological and biochemical responses.

## 2. Results

### 2.1. Morphological Measurements and Leaf Chlorophyll Content Determination (SPAD)

At the end of the trial period, the highest reduction in leaf area was observed in C35 citrange (−54.7%) and *C. volkameriana* (−43.9%) at 66%, while Carrizo citrange and *C. volkameriana* had the highest decrement at 50% Et_0_ (Table 1). Water stress significantly increased the leaf area of Bitters with respect to control both at 66% and 50% Et_0_, while Furr significantly increased the leaf area at 50% Et_0_.

A significant reduction in shoot length was noticed in C35 citrange, Furr citrandarin and *C. volkameriana* both at 66% and 50% Et_0_, while the others did not exhibit statistical difference (Table 1).

Generally, water stress significantly reduced root length with respect to the control values, even if no statistical difference was detected in Carpenter, Furr, *C. macrophylla* and *C. volkameriana* both at 66% and 50% Et_0_ (Table 1). Overall, it was noted that C35 citrange, Swingle citrumelo and Bitters citrandarin had the highest reduction in root length. Drought reduced the shoot/root dry weight of all rootstocks with respect to the control values, except for Carpenter, Furr and *C. macrophylla* which did not show statistical differences in response to water stress (Table 1).

**Figure 1:**
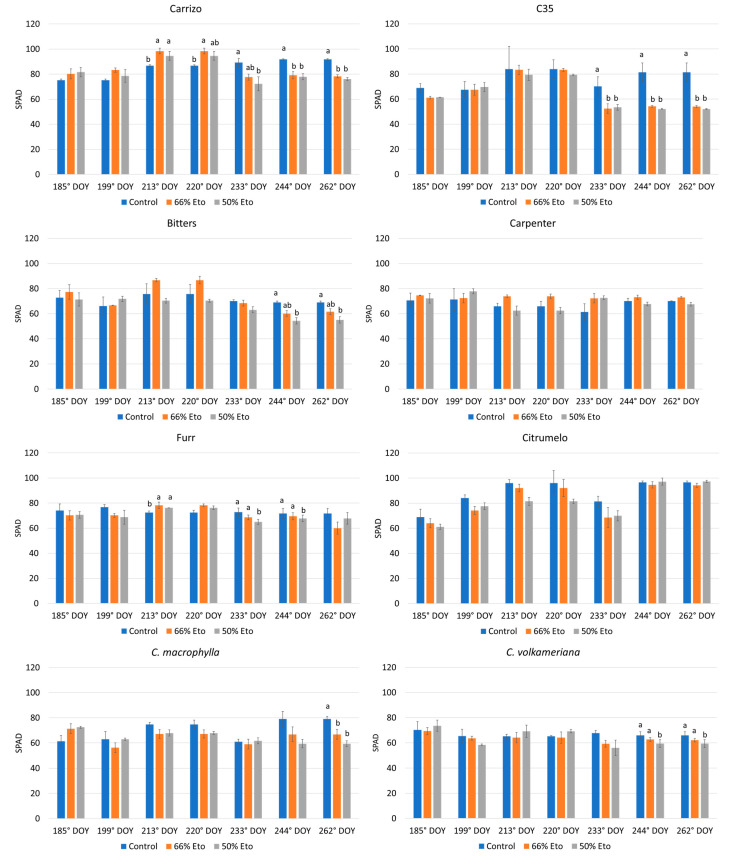
Chlorophyll index (SPAD) of 8 rootstocks subjected to water stress from 185 to 262 days of the year (DOYs). Means ± standard deviation of the three treatments analyzed in triplicate are reported. Values without letters have no significant differences according to Fisher′s LSD procedure at a 95% confidence level.

A different response of the SPAD index was exhibited by the studied rootstocks during the experiment (Figure 1). In particular, a significant decrease in SPAD values compared to the control was noted in Carrizo and C35 citranges at both water shortage levels, especially after 233° DOYs. C. macrophylla exhibited a high sensitivity to both water stress imposed only at 262° DOYs (Figure 1), while *C. volkameriana* revealed a significant decrement in SPAD values after 244° DOYs at 50% Et_0_. No statistical differences were noted in Bitters, Carpenter, Furr and citrumelo compared to the control in both treatments.

### 2.2. Plant Water Status and Physiological Monitoring of the Plants

**Figure 2:**
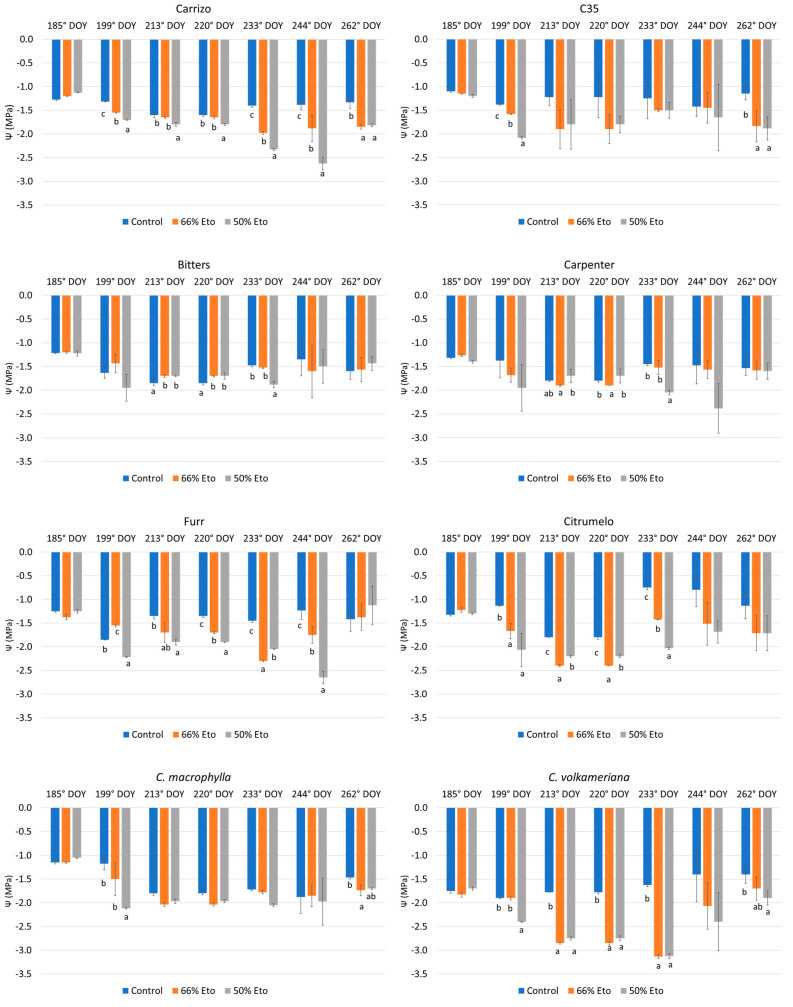
Xylem water potential (Ψ, MPa) determined in 8 rootstocks subjected to water stress from 185 to 262 days of the year (DOYs). Means ± standard deviation of the three treatments analyzed in triplicate are reported. Values without letters have no significant differences according to Fisher’s LSD procedure at a 95 % confidence level.

Generally, during experiments, stem xylem water potential decreased with increasing water stress, especially in Carrizo and C35 citranges and *C. volkameriana* (Figure 2), with the latter exhibiting the lowest values. In detail, it was observed that Carrizo decreased the Ψ values from 199° DOYs until the end of the trial, whereas C35 significantly reduced the values with respect to the control only at the last detection (262° DOYs) at 66% and 50% Et_0_ (59% and 64% less, respectively). During the trial Furr, citrumelo and *C. volkameriana* showed a sensitive response to water stress, whilst Bitters, Carpenter and *C. macrophylla* maintained similar values in all treatments (Figure 2).

**Figure 3:**
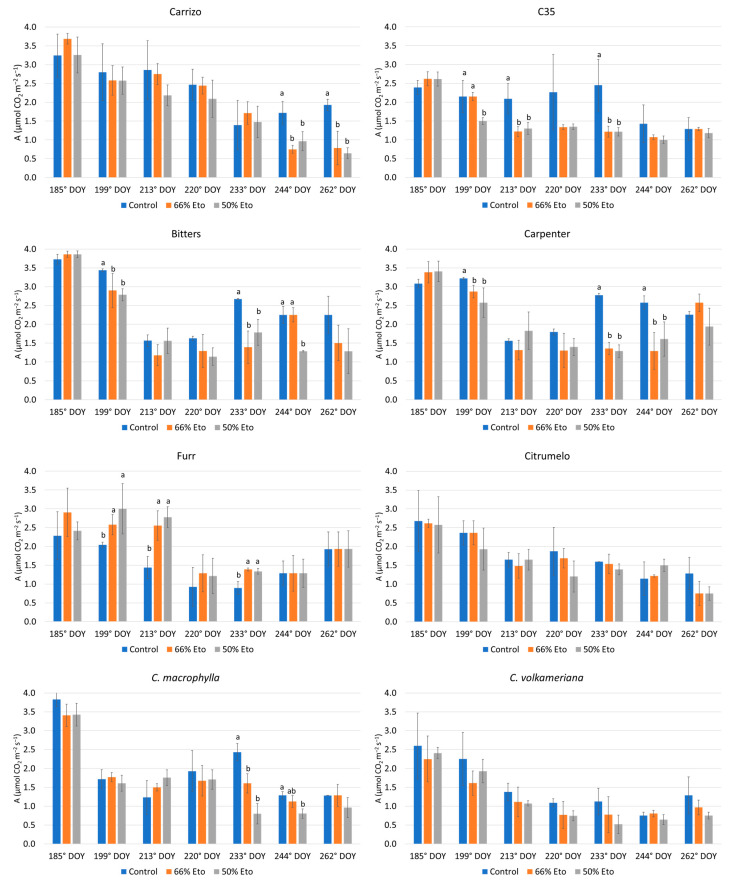
Net photosynthesis values (A, μmol CO_2_ m^−2^ s^−1^) of 8 rootstocks subjected to water stress from 185 to 262 days of the year (DOYs). Means ± standard deviation of the three treatments analyzed in triplicate are reported. Values without letters have no significant differences according to Fisher’s LSD procedure at a 95% confidence level.

Overall, the net photosynthetic rate (A) monitored during the experiment showed a slight decrease in all rootstocks (Figure 3). Carrizo citrange exhibited the highest A reduction with respect to the control at the end of the trial, precisely at 244° DOYs (−56.3% and −43.8%, respectively, for 66% and 50% Et_0_) and at 262° DOYs (−59.3% and −66.6%, respectively, for 66% and 50% Et_0_). The genotypes C35, Bitters, Carpenter, Furr and *C. macrophylla* exhibited some statistical differences during the trial, even if no significant difference was observed at the end of the experiment in the two stressed treatments. Generally, transpiration rate (E) lowered during the experiment in all treatments (Figure S1). Just at the end of the experiment, both treatments induced an increment of E in C35 and Citrumelo (Figure 3), while Bitters and *C. volkameriana* exhibited a reduction in E at the end of the trial with respect to the control in both stresses. Regarding the stomatal conductance (gs) a significant decrement of gs was observed in C35 citrange (−24.6% and −54.8%, respectively, for 66% and 50% Et_0_) and in *C. volkameriana* (−66.7% and −50.0%, respectively, for 66% and 50% Et0) in response to water stress (Figure 3) at the end of the trial (Figure S1). It was noted that Carpenter (−66.7), Furr (−60.0%) and Citrumelo (−33.3%) presented a significant reduction in the gs value at 50% Et_0_, whereas *C. macrophylla* showed an increase at 66% and 50% Et_0_ (Figure 3).

A significant reduction in the maximum potential quantum yield of PS2 (Fv/Fm) was recorded in C35 and Citrumelo at 66% Et_0_ (Figure S2), while a significant increase was noted in Bitters with respect to the control in both stresses (5.3% and 1.2%, respectively, for 66% and 50% Et_0_).

### 2.3. Proline Accumulation, Malondialdehyde Determination and Antioxidant Enzyme Activities

**Figure 4:**
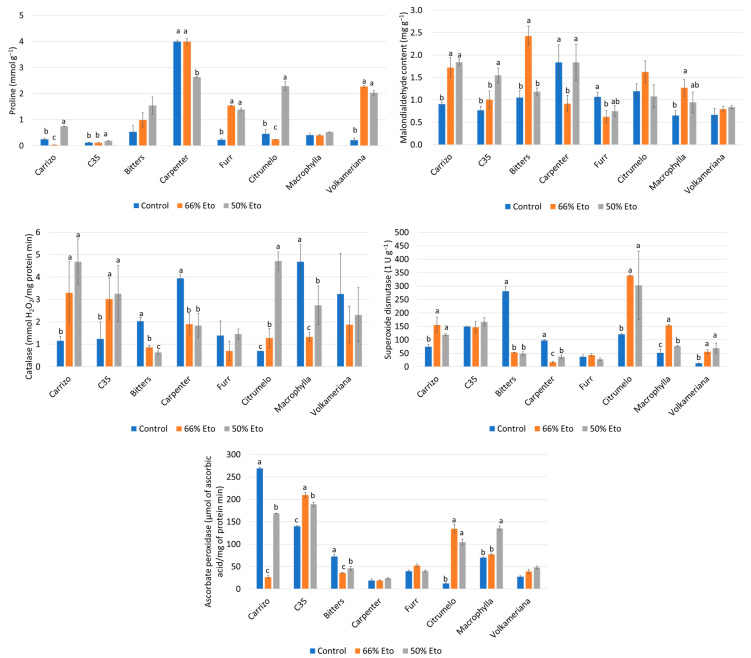
Proline (mmol g^−1^) and malondialdehyde content (MDA, mg g^−1^), catalase (mmol H_2_O_2_/mg protein min), superoxide dismutase (SOD, 1 U g^−1^), ascorbate peroxidase (APX, μmol of ascorbic acid/mg of protein min) measured at the end of the trial in the leaves of 8 rootstocks subjected by water stress. Means ± standard deviation of the three treatments analyzed in triplicate are reported. Values without letters have no significant differences according to Fisher’s LSD procedure at a 95 % confidence level.

At the end of the experiment, the accumulation of proline and malondialdehyde in the leaves and the antioxidant enzyme activities of the leaves were measured (Figure 4).

A significant increase in proline, with respect to the control, was recorded in Furr and *C. volkameriana* both at 66% and 50% Et_0_ (Figure 4). Carpenter showed a significant reduction only at 50% Et_0_ with respect to the control (−34.0%), while Carrizo (−85.0%) and Citrumelo (−47.0%) showed a reduction at 66% Et_0_. Finally, Carrizo, Citrumelo and C35 exhibited an increase with respect to the control only at 50% Et_0_. No statistical differences among stressed and not-stressed plants were recorded in Bitters and *C. macrophylla*.

As regards lipid peroxidation, Citrumelo and C. volkameriana showed no statistical differences at both stressed conditions compared to the control, whereas Carpenter and Furr reduced the MDA content at 66% Et_0_ (−50.1 and −41.8%, respectively), but displayed similar values to the control at 50% Et_0_. On the contrary, Carrizo citrange showed a significant increase in MDA values at both stresses (90.3 and 103.3%, for 66 and 50% Et_0_), while C35 citrange exhibited a significant accumulation only at the maximum stress (102.1%) (Figure 4). Finally, Bitters and *C. macrophylla* showed an increased level of lipid peroxidation only at 66% Et_0_ (130.8 and 94.6%, respectively).

As regards antioxidant enzymatic activities, different trends in each rootstock genotype were observed. All activities always showed similar values to those recorded in the control only in Furr, whereas a significant reduction was observed in Bitters (−68.6% for CAT, −36.7% for APX and 82.5% for SOD). In Citrumelo, all three enzymatic activities were higher, with respect to the control at both levels of stress, while in *C. macrophylla,* an increase was recorded only at higher levels of stress (93.7% for APX and 48.2% for SOD). In Carrizo, significant increases, with respect to the control, for CAT and SOD activities were recorded (305 and 60.6%, respectively), while a reduction was registered for APX values (−90.1 and −37.2% at 66 and 50% Et_0_). On the contrary, C35 showed an increase with respect to the control for CAT and APX. Carpenter reduced CAT activities but increased SOD at all levels of stress. Finally, *C. volkameriana* exhibited only an increase in SOD activities at both levels of stress.

### 2.4. Hormone Analyses

**Figure 5:**
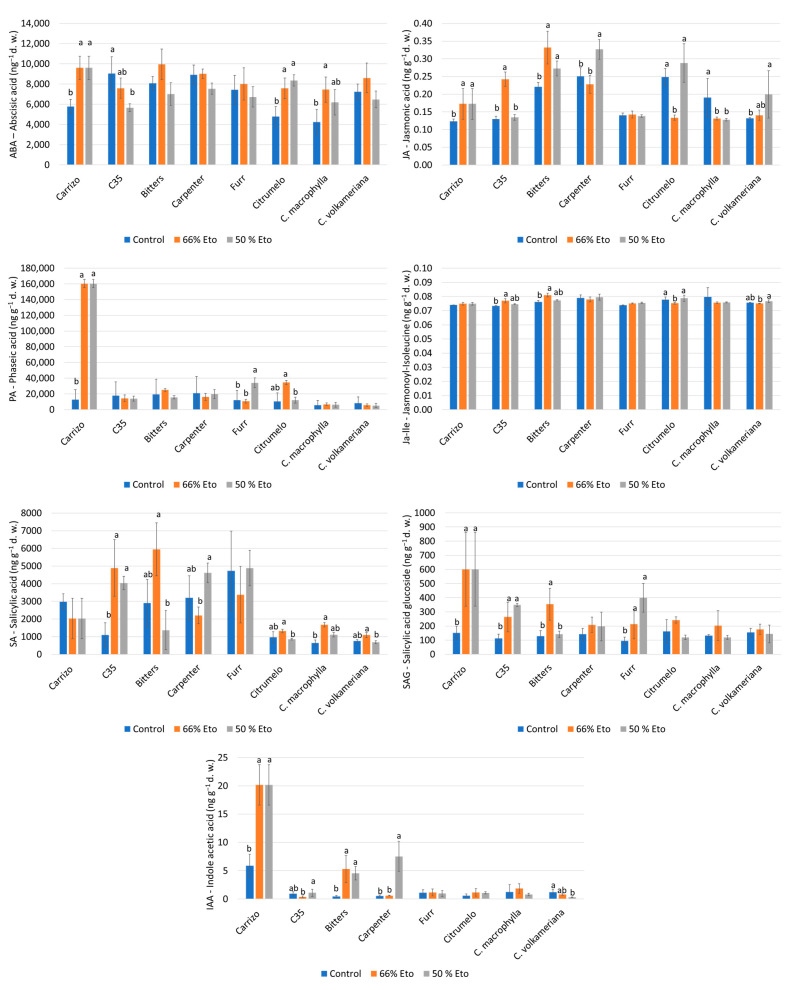
Hormones measured at the end of the trial in leaves of 8 rootstocks subjected to water stress. ABA: abscisic acid (ng g^−1^ dry weight); JA: jasmonic acid (ng g^−1^ d. w.); SA: Salicylic acid (ng g^−1^ d. w.); SAG: salicylic acid glucoside (ng g^−1^ d. w.); IAA: indole acetic acid (ng g^−1^ d. w.); Ja-lle: jasmonoyl–isoleucine (ng g^−1^ d. w.); and PA: phaseic acid (ng g^−1^ d. w.). Means ± standard deviation of the three treatments analyzed in triplicate are reported. Values without letters have no significant differences according to Fisher′s LSD procedure at a 95 % confidence level.

Hormones were quantified in the leaves of the eight rootstocks at the end of the experiment (Figure 5). At the end of the experiment, an accumulation of abscisic acid (ABA) was recorded in Carrizo, Citrumelo and *C. macrophylla*, even if the latter did not show statistical differences at 50% Et_0_ compared to the control. Bitters, Carpenter and Furr citrandarins and *C. volkameriana* presented a similar trend to the control, while a progressive decrease in C35 was recorded at increasing water stress levels. A significant increase in the jasmonic acid (JA) value was noted in Carrizo and Bitters at 66% and 50% Et_0_. Carpenter and *C. volkameriana* exhibited an increase only at 50% Et_0_, although the latter did not show statistical differences compared to the control. A decrement in JA accumulation in *C. macrophylla* was noticed at 66% and 50% Et_0_. Carrizo exhibited a significant accumulation in phaseic acid (PA) at both levels of water stress, while Furr increased the PA content at 50% Et_0_. Regarding the jaisoleucine (JA-lle) hormone, a similar trend with respect to the control was observed in all rootstocks except in Citrumelo. C35 citrange showed a significant accumulation of salicylic acid (SA) at both levels of water stress, while Bitters had the highest accumulation at 66% Et_0_, even if no statistical difference was observed compared to the control. Carrizo, C35 and Furr had the highest accumulation of salicylic acid glucoside (SAG), the inactive form of SA (as glucosides of SA), whereas Bitters exhibited a significant accumulation at 66% Et_0_. A progressive increase in indoleacetic acid (IAA) was recorded in Carrizo, Bitters and Carpenter at 66% and 50% Et_0_, while Furr, *C. macrophylla* and *C. volkameriana* showed a light decrement of the IAA value, although no statistical differences were recorded respect with the control.

### 2.5. Principal Component Analysis and Pearson’s Correlation Test

**Figure 6:**
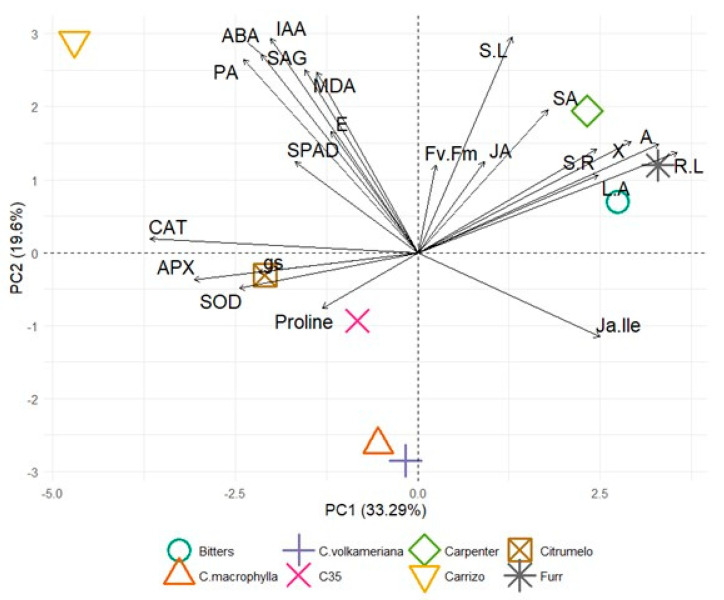
Principal component analysis (PCA) showing the distribution of the eight rootstocks subjected to water stress. The parameters measured were xylem water potential (Ҳ), net photosynthesis (A), transpiration (E), stomatal conductance (gs), chlorophyll fluorescence (Fv/Fm), chlorophyll meter (SPAD), catalase (CAT), superoxide dismutase (SOD), ascorbate peroxidase (APX), malondialdehyde content (MDA), proline content (Proline), leaf area (L-A), root length (R-L), shoot length (S-L) shoot/root ratio (S/R), abscisic acid (ABA), jasmonic acid (JA), salicylic acid (SA), salicylic acid glucoside (SAG), indole acetic acid (IAA), jasmonoyl–isoleucine (Ja-lle), and phaseic acid (PA).

A principal component analysis (PCA) on the morphological, physiological, chemical and hormone parameters was performed to assess the differential response of several rootstocks under water stress (Figure 6). The first two principal components accounted for 52.89% of the total variance (PC1 = 33.29%; PC2 = 19.6%). The main variables contributing to PC1 were A (0.310), followed by root length (0.333) and Ψ (0.273), while PC1 was negatively related with catalase activity (−0.343) and APX (−0.286). The PC2 was highly correlated with shoot length (0.360), IAA (0.358), ABA (0.330) and PA (0.323).

It was noticed that Furr (0.430), Bitters (0.360) and Carpenter (0.303) citrandarins were highly correlated with PC1, while Carrizo (−0.615) exhibited a negative relation with PC1. In fact, it was observed that citrandarins were greatly affected by physiological measurement, specifically by the net photosynthetic rate and xylem water potential (Figure 2, Figure 3 and Figure S1). On the contrary, Carrizo showed a significant reduction in root length (Table 1) and a great increase in catalase activity (Figure 4). Carrizo citrange (0.495) showed a positive correlation with PC2, while *C. volkameriana* (−0.485) and *C. macrophylla* (−0.448) had a negative correlation with PC2. Carrizo exhibited a significant accumulation in ABA (Figure 5), while *C. volkameriana* exhibited a reduction in shoot length at 66% and 50% Et_0_ (Table 1).

**Figure 7:**
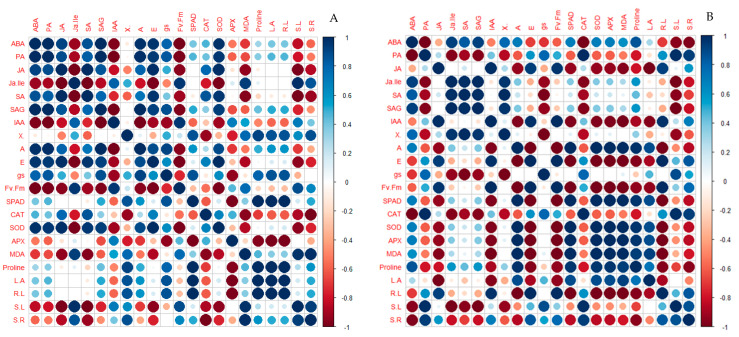

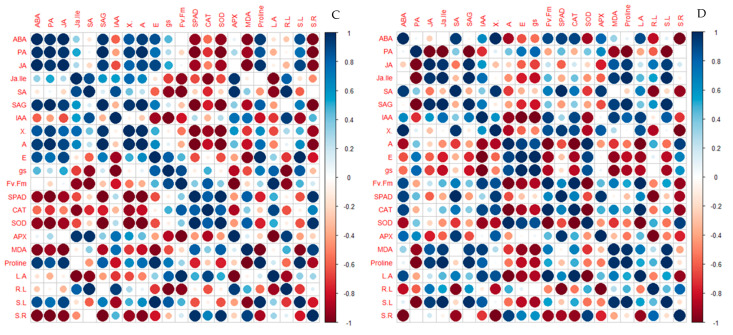

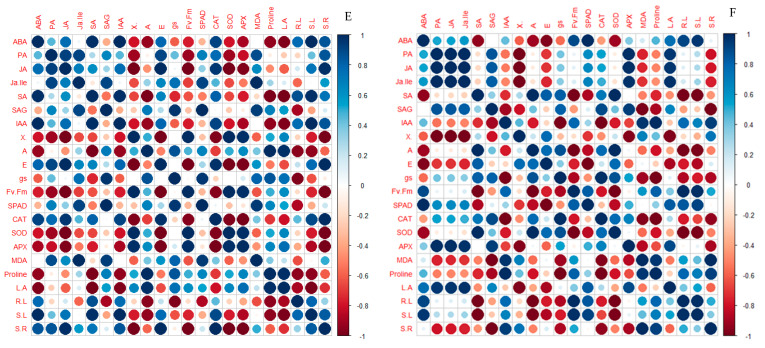

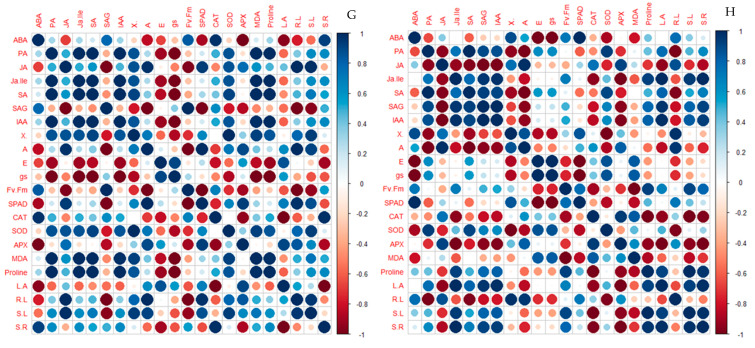
Pearson’s correlation coefficients among the parameters measured in the eight rootstocks subjected to water stress (Carrizo (**A**), C35 (**B**), Bitters (**C**), Carpenter (**D**), Furr (**E**), Citrumelo (**F**), *C. macrophylla* (**G**) and *C. volkameriana* (**H**)). The parameters measured were xylem water potential (Ҳ), net photosynthesis (A), transpiration (E) and stomatal conductance (gs), chlorophyll fluorescence (Fv/Fm), chlorophyll meter (SPAD), catalase (CAT), superoxide dismutase (SOD), ascorbate peroxidase (APX), malondialdehyde content (MDA), proline content (Proline), leaf area (L-A), root length (R-L), shoot length (S-L) shoot/root ratio (S/R), abscisic acid (ABA), jasmonic acid (JA), salicylic acid (SA), salicylic acid glucoside (SAG), indole acetic acid (IAA) and jasmonoyl-isoleucine (Ja-lle), Phaseic acid (PA).

Correlation coefficients of morphological, physiological, chemical and hormone parameters are shown in Figure 7. In Carrizo citrange, the ABA value was positively correlated with PA (0.99), SA (0.87) and SAG (0.99), while it presented a negative correlation with IAA (−0.98) and Jalle (−0.92). PA exhibited a positive relationship with SA (0.83) while PA, JA, SA and SAG were negatively related with IAA (−0.99, −0.82, −0.74, −0.99, respectively) and with Jalle (−0.87, −0.99, −0.99 and −0.85). Water xylem potential was negatively related with JA (−0.50) and SA (−0.59). Net photosynthesis was positively correlated with all quantified hormones except Jalle (−0.81) and IAA (−0.99). The same trend was observed in the transpiration value, while gs exhibited a negative correlation with the IAA hormone (−0.91). It was observed that fluorescence had a high relationship only with hormones Jalle (0.95) and IAA (0.95). Catalase exhibited a high relationship with JA (0.79) and SA (0.85), while a strongly negative correlation was observed with Jalle, xylem water potential, MDA, shoot length and shoot/root ratio. SOD was negatively related with Jalle (−0.99), IAA (−0.98), Fv/Fm (−0.99), MDA (−0.86), shoot length (−0.94) and shoot/root ratio (−0.76). APX was mostly negatively related with gs, SPAD, leaf area and root length. Furthermore, MDA was positively related with Jalle, xylem water potential, Fv/Fm, shoot length and shoot/root ratio. Proline showed a strong negative correlation only with APX activity (−0.97). Furthermore, it was observed that shoot length and shoot/root ratio were negatively linked to all hormones except Jalle.

In C35 citrange, it was observed that abscisic acid was highly negatively correlated with PA (−0.99), CAT (−0.99), root length (−0.70), shoot length (−0.96) and shoot/root ratio (−0.97). PA was positively correlated with gs, CAT, shoot length and shoot/root ratio. In addition, JA was negatively associated with all enzyme activities, MDA, proline and leaf area. It was observed that Jalle, SA and SAG exhibited the same negative trend with gs, CAT, shoot length and shoot/root ratio. IAA was negatively correlated with SPAD (−0.97), enzyme activity, SOD (−0.97), APX (−0.97), MDA (−0.88), proline content (−0.97) and leaf area (−0.97). It was recorded that net photosynthesis, transpiration and fluorescence showed an inverse trend; the A value was highly correlated with SPAD, SOD, APX, MDA, proline and leaf area, while the E value and Fv/Fm were negatively related to these parameters. It was found that stomatal conductance was positively related to PA hormone (0.79), CAT (0.79) and shoot length (0.89). Proline content was negatively correlated with PA (−0.79), Ja (−0.80), IAA (−0.88), E (−0.96), Fv/Fm (−0.93), root length (−0.97), shoot length (−0.67) and shoot/root ratio (−0.95).

In the rootstock Bitters, it was observed that all hormones were correlated with each other except IAA which was negatively linked to ABA (−0.59), PA (−0.41), JA (−0.64) and SAG (−0.35). Furthermore, a very highly negative correlation was recorded between the hormones ABA, PA, JA, Jalle and SA and the values of SPAD and antioxidant activity, i.e., CAT and SOD. Xylem water potential and net photosynthesis exhibited the same negative trend with SPAD, CAT, SOD, MDA, leaf area and shoot/root ratio. The E value was negatively related only with IAA hormone (−0.90), leaf MDA content (−0.96), root length (−0.70) and shoot/root ratio (−0.79). Stomatal conductance and fluorescence showed the same negative trend with Jalle, IAA, APX and root length. SPAD values measured in leaves exhibited a positive correlation with enzyme activity (i.e., CAT and SOD), MDA and shoot/root ratio.

In Carpenter, it was recorded that ABA, JA, Jalle, SA, SAG and IAA were negatively correlated with physiological analyses, particularly with A, E and gs values. IAA was strongly negatively correlated with SOD (−0.92) and positively correlated with all other enzymatic measures. A negative correlation was recorded between xylem water potential and physiological measures, while Ψ was correlated with Fv/Fm.

Overall, it was observed in Furr that all hormones were positively correlated with each other. Water xylem potential was negatively correlated with ABA (−0.80), PA (−0.90), JA (−0.99), Jalle (−0.65), SA (−0.66), SAG (−0.26) and IAA (−0.80). All hormones were negatively correlated with SOD and APX. ABA, SA and IAA exhibited a large negative correlation with proline and leaf area. Transpiration was negatively correlated with water xylem potential (−1.00), fluorescence (−0.99), SOD (−0.99) and APX (−0.99). MDA had a positive relation with PA (0.89), Jalle (0.99), SAG (0.93) and SPAD (0.94).

Regarding Citrumelo, ABA showed a negative correlation with SA (−0.73). Abscisic acid was negatively correlated with all physiological activities, and it was highly negatively correlated with SOD (−0.90). PA, JA, Jalle and SAG were negatively correlated with MDA and Proline, while IAA showed a positive relationship with these measures. The A value was negatively related with MDA, Proline, leaf area, root and shoot length and shoot/root ratio. Furthermore, it was observed that fluorescence and SPAD had a negative correlation with A and E values.

Plants of *C. macrophylla* showed a positive correlation between hormones. ABA was negatively related to A (−0.83), E (−0.67), SPAD (−0.88), APX (−0.99), leaf area (−0.95), root length (−0.77) and shoot length (−0.55). PA, Jalle and Sa were negatively linked with E and gs values. Indeed, the xylem water potential was positively correlated with all parameters except A, E, gs and Fv/Fm.

In *C. volkameriana* was recorded that ABA exhibited a negative correlation with E, gs, SOD and MDA. Jasmonic acid was negatively related to SOD (−0.75), proline (−0.71), leaf area (−0.94) and shoot/root ratio (−0.74). Jalle was negatively related to catalase (−0.94) and ascorbate peroxidase (−0.99), while it was positively related to proline (0.92), leaf area (0.99) and shoot/root ratio (0.94). Photosynthesis was negatively linked to all hormones except JA and ABA. In addition, the A value was strongly negatively linked to SOD activity and leaf area.

## 3. Materials and Methods

### 3.1. Plant Material and Water Treatments

Rootstocks used for the trials were obtained by seeds. Seeds were extracted from mature fruits of eight citrus rootstocks including *Citrus macrophylla* Wester, *C. volkameriana* Ten. & Pasq., Swingle citrumelo (*C. paradisi* Macf. × *Poncirus trifoliata* [L.] Raf.), Carrizo citrange (*C. sinensis* cv. Washington navel × *P. trifoliata*), C35 [*C. sinensis* (L.) Osb. cv. ‘Ruby’ × *P. trifoliata* (L.) Raf.] and the citrandarins Bitters (C22), Carpenter (C54), Furr (C57) (*C. sunki* × *P. trifoliata*). Seeds were sown into a pre-moistened substrate composed of peat, coconut fiber, sand and perlite (50:25:20:5). Nucellar seedlings were transplanted in pots and were irrigated three times a week (280 mL per watering shift) and fertilized using a Hoagland solution, as modified by Forner-Giner et al. [15] until the beginning of the experiment.

In total, 10 one-year-old plants in 9 L pots per treatment were selected homogeneous in size for each rootstock (i.e., 10 seedlings × 8 cultivars × 3 treatments). Water was supplied for all treatments three times a week to compensate for evapotranspiration; water volume was previously calculated by means of gravimetric water loss, determining the weight of saturated soil and field capacity [25]. Three treatments were imposed: control (280 mL per irrigation shift per pot; 100% Et_0_), 66% Et_0_ (185 mL) and 50% Et_0_ (140 mL). Air temperature and relative humidity (RH) were recorded hourly in the greenhouse by a datalogger (Elitech RC-51 H USB, London, UK) during the experiment from 185 to 262 days of the year (DOYs) in 2020.

### 3.2. Morphological Analyses and Leaf Chlorophyll Content Determination (SPAD)

Immediately after the end of the experiment, all the ten plants per rootstock and treatment were used for morphological analyses. Leaf area (m^2^), shoot (cm) and root length (cm) were measured after harvesting, as described by Modica et al. [26]. Leaf chlorophyll index was measured using SPAD chlorophyll meter (Minolta Co., Osaka, Japan), as reported by Modica et al. [26].

### 3.3. Plant Water Status and Physiological Monitoring of the Plants

Physiological parameters were measured fortnightly from 185 to 262 DOYs (i.e., 7 times during the experimental period), as reported by Modica et al. [26].

The xylem water potential Ψ (MPa) was monitored using a Scholander pressure chamber (Model 600, PMS Instrument Company, Albany, OR, USA), as reported by Vanella et al. [27].

Measurements were made on clear days, between 8:00 h and 10:00 h (solar time) on fully developed leaves (3 replicates per treatment for each rootstock).

Simultaneously, chlorophyll fluorescence (Fv/Fm) was monitored using a portable modulated pulse fluorometer (Handy Pea, Hansatech Instruments Ltd., Norfolk, UK), as reported in Modica et al. [26]. The Fv/Fm ratio, used to express the chlorophyll fluorescence, was calculated according to Schreiber et al. [28].

### 3.4. Proline and Malondialdehyde Accumulation, and Antioxidant Enzyme Activities

At the end of the trial (262 DOYs), 30 leaves per rootstock and treatment were harvested for biochemical analyses. Leaves were crushed in liquid nitrogen to obtain a fine powder. Afterwards, powdered plant tissues (0.5 g) were homogenized in 5 mL of potassium phosphate buffer 50 mM (pH 7.8), 1 mM Ethylenediaminetetraacetic acid (EDTA), 1 mM Dithiothreitol (DTT), 1% Polyvinylpyrrolidone (PVP) *w*/*v* and 1 mM Phenylmethylsulfonyl fluoride (PMSF). Samples were filtered through 3 layers of gauze, and centrifuged at 15,000× *g* for 30 min at 4 °C.

The resulting supernatant was recovered, and the total proteins were precipitated with solid (NH_4_)_2_SO_4_ at 55% saturation. Enzymatic activities were performed by using the total protein extract from leaves. Enzymatic aliquots were centrifuged at 13,000 rpm for 30 min at 4 °C, the supernatant was discarded and the pellet was dissolved in 4 mL 50 mM sodium–phosphate buffer (pH 7.0) containing 1 mM EDTA, 1% (*w*/*v*) PVP-40 (*w*/*v*) and 1 mM PMSF [29]. Ascorbate peroxidase (APX, EC 1.11.1.11) was measured according to Ushimaru et al. [30] and as described by Modica et al. [26], catalase (CAT, EC 1.11.1.6) was determined as described by Aebi [31] and Superoxide dismutase (SOD, EC 1.15.1.1) activity was examined as reported by Modica et al. [26] and according to Masia [32]. The total protein content was quantified by the Bradford [33] method, using Bovine Serum Albumin (BSA) at different concentrations as a standard curve.

Lipid peroxidation was calculated by measuring the concentrations of malondialdehyde (MDA) as reported by Modica et al. [26].

Proline was quantified spectrophotometrically using a spectrophotometer (NanoDrop 2000, Thermo Scientific, Waltham, MA, USA) following the ninhydrin method of Bates et al. [34] and modified by Khedr et al. [35]. The citrus fresh leaves (1 g) were homogenized in 3% aqueous sulphosalicylic acid and the residues were removed by centrifugation at 15,000× *g* for 10 min. The supernatant (1 mL) was mixed with 1 mL of glacial acetic acid and ninhydrin reagent in a 1:1 (*v*/*v*) ratio. The reaction mixture was incubated at 100 °C for 1 h. After extraction using toluene, the absorbance of the organic phase was measured at 520 nm. The proline concentration was determined as reported by Bates et al. [34], using D-proline at different concentrations as a standard curve.

### 3.5. Hormone Analyses

The hormones analyzed were abscisic acid (ABA), phaseic acid (PA), salicylic acid (SA), indoleacetic acid (IAA), jasmonic acid (JA) and JAIsoleucine (JA-Ile). Hormone extraction was carried out with 15 mg of freeze-dried leaves, as described in Modica et al. [26]. The solution was filtered using 0.22 μm polytetrafluoroethylene membrane syringe filters (Albet S.A., Barcelona, Spain), diluted 1:4 and directly injected into an ultraperformance LC system (Acquity SDS; Waters Corp., Milford, MA, USA) connected online to a TQS triple quadrupole mass spectrometer (Micromass Ltd., Manchester, UK) through an orthogonal Z-spray electrospray ion source. Chromatographic separations were carried out on a reversed-phase C18 column (Luna Omega Polar C18, 50 × 2.1 mm, 1.8-μm particle size; Phenomenex, Torrance, CA, USA), as described in Modica et al. [26].

Hormones were identified following their specific precursor-to-product ion transition and quantitated using an external calibration performed with injection of standard solutions of known amount.

### 3.6. Statistical Analysis

Statistical analyses were performed using STATISTICA 6.0 (Statsoft Inc., Tulsa, OK, USA). The method used was described by Forner et al. [15]. Principal component analysis (PCA) and Pearson’s correlation test were made using R software (v. 4.3.1) computing [24,36]. The results were described using the package ‘ggplot2’ [37].

## 4. Discussion

### 4.1. Morphological Analyses, Physiological Monitoring of Plant

Among various abiotic stresses, water stress is one of the major factors limiting fruit tree crop productivity, including citrus [38,39,40]. Previous studies have highlighted the importance of citrus rootstock genotype in response to water stress and to irrigation [7,9,41,42,43].

The morphology of different genotypes can influence their resistance to drought stress [37]. In our study, at higher water stress levels, Bitters and Furr increased significantly leaf area, exhibiting an adaptive tolerant behavior to drought [44,45,46]. C35 citrange showed a reduction in leaf area, in shoot and root length, assuming a susceptibility to water stress, while in a previous work this rootstock responded to a moderate water deficit treatment (75% Et_0_) with an increase in shoots length [46].

The SPAD index was frequently used to detect some plant stress symptoms, such as chlorosis and necrosis, also due to abiotic problems, including deficiency or excess of water; these damages have been associated with the degradation of chlorophyll and of the internal structures of the chloroplast, altering the photosynthetic process [10,47,48,49,50,51].

Previous studies described that SPAD values were similar to the control in Carrizo citrange plants subjected to different water stresses for 30 days [4]; in our study, no difference after 30 days was exhibited, while a significant decrease in SPAD index in Carrizo and C35 citranges compared to the control was evident just after 48 days from the beginning of both the imposed stresses (66 and 50 Et_0_). Chlorophyll degradation may be related to its oxidation as a consequence of excess ROS generated by abiotic stress [52,53] and confirmed by the accumulation of MDA as a consequence of membrane peroxidation [54].

Some citrus genotypes have the ability to counteract water deficit by allowing the plant to maintain tissue water potential and stem water content close to the unstressed level. In this way stressed plants increase water absorption or limit water loss [55]. Conversely, the reduction in xylem water potential observed in Carrizo and C35 citranges and *C. volkameriana* suggested their susceptibility to water stress. Some researchers [4,6] observed that Carrizo citrange plants subjected to 50% Et_0_ for 30 days did not show significant differences in leaf water potential compared to the control. On the contrary, in our investigation Carrizo and C35 citrange exhibited significant sensitivity to both water stresses in accordance with previous results [56]. At the end of the trial there was no statistical difference in Ψ values of Bitters, Carpenter and *C. macrophylla* with respect to the control as a response to the osmotic stress of the plants that reduced water loss by decreasing stomatal conductance; in addition, morphological modifications, especially in the root system, may contribute in order to maximize water absorption [9,57,58]. Gas exchanges revealed a decrease only after more than 60 days from the beginning of the experiment. In detail, the stomatal conductance lowered in the more severe treatment in Carpenter, Furr and citrumelo, with C35 and *C. volkameriana* the most susceptible already at 66% Et_0_. Regarding photosynthesis values, drought-treated Carrizo plants showed greater reductions in leaf photosynthesis than the other genotypes just 60 days after the start of the test, confirming previous results [59].

### 4.2. Proline and Malondialdehyde Quantification, Enxymatic Activities and Homone Analyses

The amino acid proline is accumulated in plant tissues in response to various abiotic stresses as it plays an important role against oxidative damage caused by ROS [60]. It was noticed that the proline content of Bitters and *C. macrophylla* was similar to the control, suggesting a higher tolerance to intermediate and severe water stress [57]; although, there were higher values of MDA in 66% Et_0_. Indeed, a significant accumulation of MDA was observed in Carrizo, but an increase in CAT and SOD in leaves confirmed that enzyme activities increased in response to abiotic stresses, including water deficit [61]. Overall, Furr showed a low level of lipid peroxidation, showing MDA accumulation lower (66% Et_0_) or similar (50% Et_0_) to the control, and maintaining all enzymatic activities to values similar to the control. On the contrary, Carrizo and C35 were shown to be more sensitive to water stress, as MDA accumulation increased, especially in more severe water stress, and in accordance, they also raised the antioxidant enzymes, which tried to counteract the drought stress condition. The involvement of ABA in the regulation of citrus responses to abiotic stresses, including drought is now well known [20,26,62,63]. During water stress, ABA was involved in plant survival and growth, and its increase or decrease in leaves regulates downstream signaling pathways to promote plant growth under stress conditions [64]. In this context, Carrizo exhibited a significant accumulation of ABA at 66 and 50% Et_0_ compared to the control, while Bitters, Carpenter, Furr citrandarins and *C. volkameriana* presented a similar trend to the control. Previous studies observed that JA is involved in leaf senescence but also in plant responses to water deficiency [65]. It was observed that there was a significant accumulation of JA concentrations in Carrizo and Bitters at both stress levels; it is possible to hypothesize that the accumulation of this hormone in the leaves of the genotypes is due to a sensitivity to drought stress. However, it is important to consider the involvement of JA in the triggering of downstream events, because, as previously reported, JA content was recorded as an early mediator between the perception of stress and the induction of physiological responses [66,67,68]. It was noticed that an accumulation of PA in Carrizo at both levels of water stress, confirming previous studies reporting that abiotic stresses, including water stress, result in the production of phasic acid (PA) and dehydrophasic acid (DPA) in susceptible genotypes [63]. C35 citrange exhibited an accumulation of SA, while Carrizo, C35 and Furr had the highest accumulation of SAG. It was reported that SA and its inactive form (SAG) play a key role in abiotic stress; in fact, SA accumulation has often been associated with a decrease in plant antioxidant activity [64]. Regarding IAA, a progressive accumulation was recorded in Carrizo, Bitters and Carpenter at both stress levels, while Furr, *C. macrophylla* and *C. volkameriana* presented a light decrement of the IAA value, although no statistical differences were recorded with respect to the control. Previously, it was observed that IAA did not show any change in pants affected by abiotic stress, as it is possible that water stress does not induce protein degradation and subsequent senescence through auxin stimulation [66,69,70].

### 4.3. Principal Component Analysis and Pearson’s Correlation Test

The result of PCA showed that Furr, Bitters and Carpenter citrandarins were related with PC1 and especially physiological (i.e., net photosynthesis and xylem water potential) and morphological measurement demonstrating their positive behavior in response to water stresses. Carrizo citrange contrarily exhibited a negative relation with PC1 due to a reduction in the root system and an increase in enzymatic activity. Indeed, Carrizo citrange showed a positive relationship with abscisic acid confirming its susceptibility to abiotic stress [26,56,64].

To reveal the effects of water stress on different rootstocks, Pearson correlation analysis was performed between different parameters. Drought caused the production and the accumulation of abscisic acid in roots and leaves [37]. Previous researchers reported that reactive oxygen species (ROS) were involved in stress-induced ABA accumulation [71]. In our results, ABA exhibited a negative correlation with APX and MDA in the plant of Carrizo citrange, while it was negatively related with CAT in leaves of C35 citrange. Indeed, ABA presented a positive correlation with proline, leaf area and root length in the plants of Carrizo and Citrumelo. On the other hand, plants develop other mechanisms to resist drought, such as increased root development or reduced leaf mass [37,72]. Furthermore, some researchers reported that citrus rootstocks respond to drought stress, showing differences in root distribution, water uptake efficiency and root hydraulic conductivities [37].

IAA showed a negative correlation with antioxidant activity in Furr, Citrumelo and *C. volkameriana* rootstocks. This could be explained because indole-3-acetic acid, the most common natural auxin, is positively involved in stress responses, as demonstrated in model plants [73]. Furthermore, these rootstocks showed an accumulation of proline, maintaining the membrane lipid peroxidation unchanged. In fact, it was observed that the MDA values were negatively correlated with the antioxidant activity, especially in the leaves of Furr, Citrumelo and *C. volkameriana*. Moreover, IAA showed a high negative correlation with morphological parameters including leaf area in Carrizo and C35 citrange. ABA measured in Carrizo was positively correlated with leaf area and root length, while in Bitters, it was linked with shoot length and in Citrumelo and *C. macrophylla,* it was correlated with all morphological parameters. Among these, Bitters is reported to be a dwarf citrus rootstock with a lowered canopy volume [74,75]. Previous researchers reported that dwarfing rootstocks had lower quantities of growth-promoting hormones, i.e., GA, IAA and cytokinins, while they accumulated a higher level of ABA in plant tissue [76]. These hormones play a key role in growth; they are positively correlated with cytokinin levels, but conversely, a negative correlation was found between IAA and plant vigor [76]. Furthermore, a negative correlation between MDA content and proline measured in leaves was recorded, mostly in Citrumelo and *C. volkameriana*, and less in Bitters, Carpenter and Furr. These osmolytes are accumulated in plants and they are considered biochemical markers of water stress levels in citrus [42]. Furthermore, the accumulation of proline is often caused by the activation of proline biosynthesis, but also by the inactivation of proline degradation caused by abiotic stress [43]. JA and its derivatives modulated several physiological processes related to plant growth, stomatal opening and nutrient uptake; indeed, they help in improving the abiotic stress tolerance in different plant species [77,78]. Previous studies showed that SA has been identified as an important compound in plant tolerance to multiple abiotic stresses because it inhibited the activity of the enzyme ACC (1-aminocyclopropane-1-carboxylic acid) synthase, preventing the formation of ethylene and the alteration of physiological processes [79,80,81]. Therefore, a positive correlation between SA and net photosynthesis was recorded in Carrizo, Bitters, Citrumelo and *C. macrophylla*. Regarding transpiration, it was observed that Carpenter, Citrumelo and *C. macrophylla* plants showed a negative correlation between the E value and the accumulation of ABA, PA, JA, Jalle and SA.

## 5. Conclusions

Water stress differently affected the investigated citrus rootstocks. Some genotypes, i.e., Bitters and Furr, exhibited a morphological strategy (e.g., increment in leaf area) aimed at reducing water consumption in drought conditions. Chlorophyll degradation was recorded in Carrizo and C35 citrange at both water shortage levels. In addition, Carrizo recorded a decrease in physiological measurements, especially net photosynthesis. Water stress also caused a decrease in xylem water potential in Carrizo, C35 citrange and *C. volkameriana*. Overall, the results of enzymatic and hormonal analyses pointed out that Bitters and Furr may be considered drought-tolerant rootstocks, while Carrizo and C35 showed a susceptible response to water stress.

## Figures and Tables

**Table 1:** Values of morphological parameters measured on 8 citrus rootstocks subjected for 78 days to different water irrigation treatments. Values followed by the same lowercase letter, within the same row, are not significantly different according to Fisher’s LSD procedure at a 95% confidence level.

	Leaf Area (m^2^)			Shoot Length (cm)			Root Length (cm)			Shoot to Root Ratio (g g^−1^)		
	Control	66% Et_0_	50% Et_0_	Control	66% Et_0_	50% Et_0_	Control	66% Et_0_	50% Et_0_	Control	66% Et_0_	50% Et_0_
**Carrizo**	3.18 ^a^	2.40 ^b^	1.62 ^c^	410.67	311.00	279.33	40.00 ^a^	38.67 ^a^	28.00 ^b^	1.80 ^a^	1.44 ^b^	1.43 ^b^
**C35**	4.44 ^a^	2.01 ^b^	3.20 ^b^	343.17 ^a^	235.67 ^b^	239.63 ^b^	40.33 ^a^	31.33 ^b^	31.50 ^b^	1.70 ^a^	1.47 ^b^	0.93 ^c^
**Bitters**	1.07 ^b^	3.47 ^a^	4.01 ^a^	397.00	396.50	384.13	45.67 ^a^	36.33 ^b^	36.75 ^b^	2.20 ^a^	1.80 ^b^	1.76 ^b^
**Carpenter**	1.82	2.02	2.09	464.67	372.90	353.75	45.33	41.33	36.75	2.10	2.43	1.44
**Furr**	2.47 ^b^	2.97 ^b^	3.70 ^a^	400.81 ^a^	287.67 ^b^	256.34 ^b^	39.00	40.00	39.33	2.00	2.25	2.42
**Citrumelo**	3.29 ^a^	2.28 ^ab^	1.84 ^b^	263.00	260.50	283.00	40.33 ^a^	34.50 ^b^	32.00 ^b^	1.50 ^a^	1.44 ^b^	0.88 ^c^
***C. macrophylla***	1.7 ^a^	1.74 ^a^	0.83 ^b^	252.17	191.67	150.83	33.00	31.00	30.67	1.60	1.41	1.46
***C. volkameriana***	3.9 ^a^	2.19 ^b^	2.07 ^b^	225.33 ^a^	186.83 ^b^	170.33 ^b^	34.33	30.67	29.67	3.00 ^a^	1.24 ^b^	1.23 ^b^

## Data Availability

The original contributions presented in the study are included in the article/Supplementary Materials, further inquiries can be directed to the corresponding authors.

## Supplementary Materials

The following supporting information can be downloaded at https://www.mdpi.com/article/10.3390/plants14050773/s1. Figure S1: Transpiration rate (E, mmol H_2_O m^−2^ s^−1^) and stomatal conductance (gs, μmol H_2_O m^−2^ s^−1^) of 8 rootstocks subjected by water stress from 185 to 262 day of the year (DOY). Means ± standard deviation of the three treatments analyzed in triplicate are reported. Values without letters have no significant differences according to Fisher’s LSD procedure at 95% confidence level.; Figure S2: Chlorophyll fluorescence (Fv/Fm) determined in 8 rootstocks subjected by water stress from 185 to 262 day of the year (DOY). Means ± standard deviation of the three treatments analyzed in triplicate are reported. Values without letters have no significant differences according to Fisher’s LSD procedure at 95 % confidence level.
