# Supplementary material for: Response to Water Stress of Eight Novel and Widely Spread Citrus Rootstocks
Source: Plants (Basel). 2025 Mar 3;14(5):773. doi: 10.3390/plants14050773 (PMC11901693; doi:10.3390/plants14050773)
Supplement: Supplementary file 1 [file plants-14-00773-s001.zip › plants-3490493-supplementary.pdf]

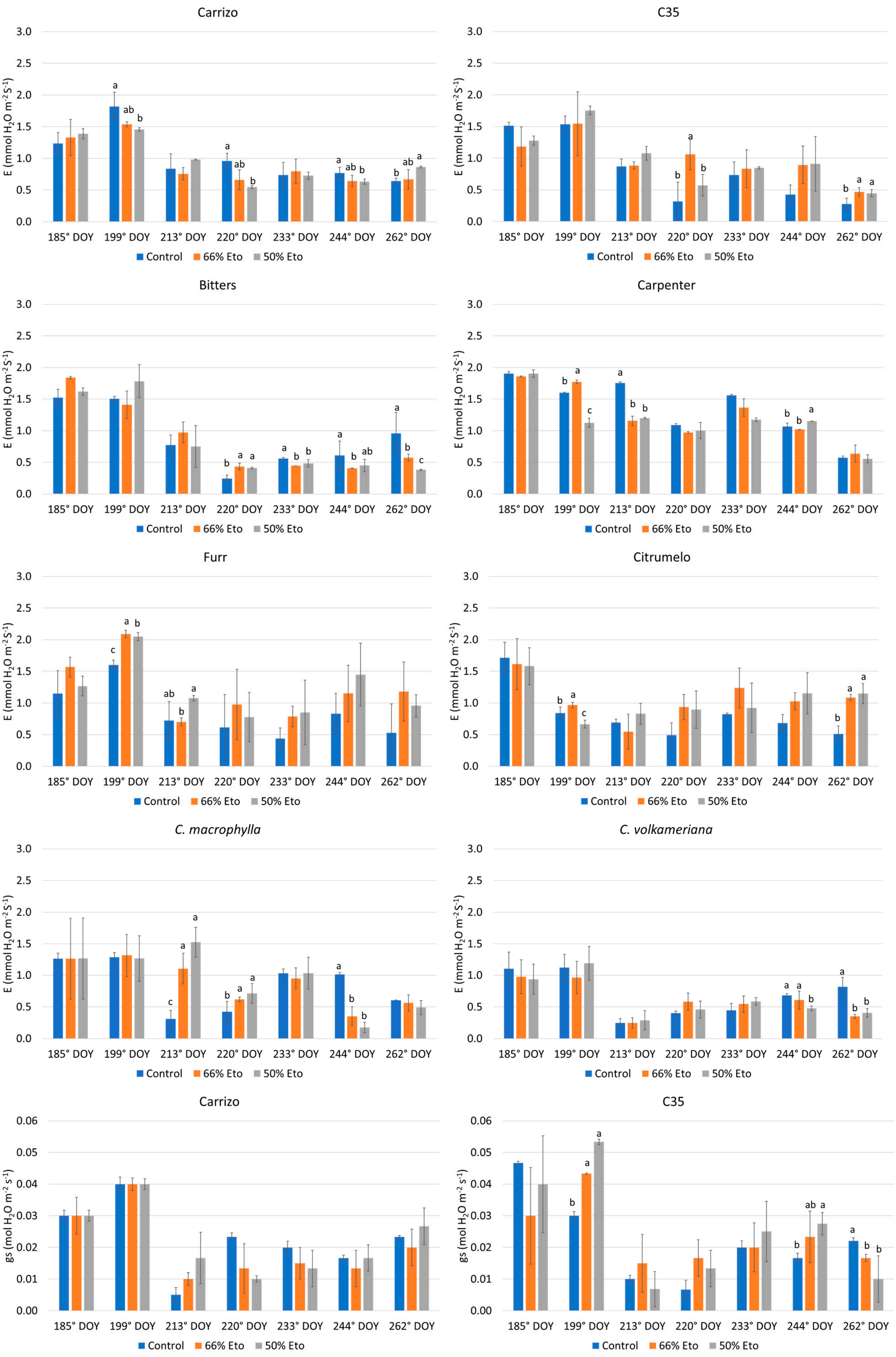
Carrizo
C35
Bitters
Carpenter
Furr
Citrumelo
C. macrophylla
C. volkameriana
Carrizo
C35
E (mmol H_2O $m^{-2}S^{-1}$)
gs (mol H_2O m^{-2} s^{-1})
0.0 0.5 1.0 1.5 2.0 2.5 3.0
0.00 0.01 0.02 0.03 0.04 0.05 0.06
185° DOY 199° DOY 213° DOY 220° DOY 233° DOY 244° DOY 262° DOY
Control 66% Eto 50% Eto

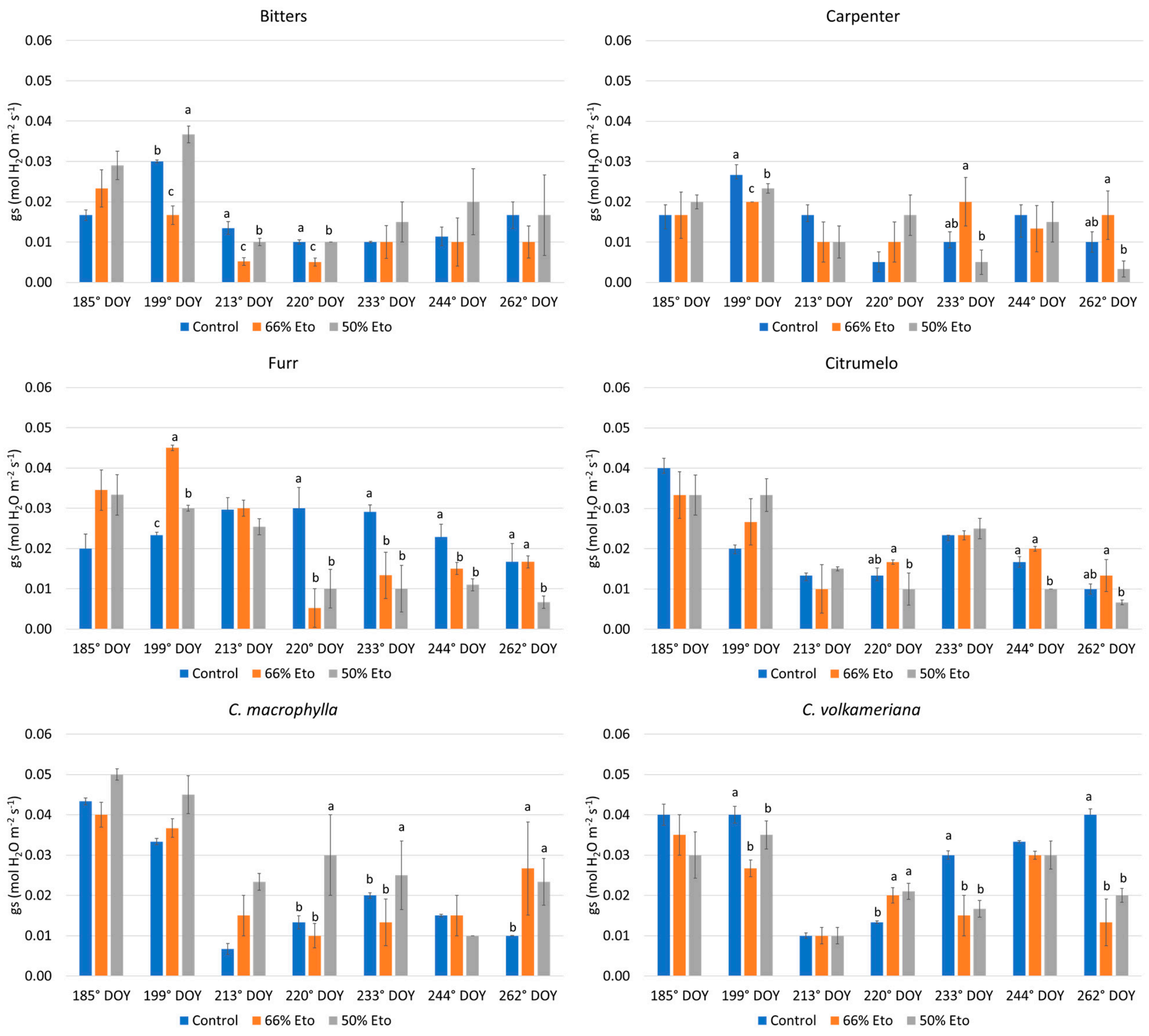

Figure S1. Transpiration rate (E, mmol H_2O m^{-2} s^{-1}) and stomatal conductance (gs, µmol H_2O m^{-2} s^{-1}) of 8 rootstocks subjected by water stress from 185 to 262 day of the year (DOY). Means ± standard deviation of the three treatments analyzed in triplicate are reported. Values without letters have no significant differences according to Fisher's LSD procedure at 95% confidence level.

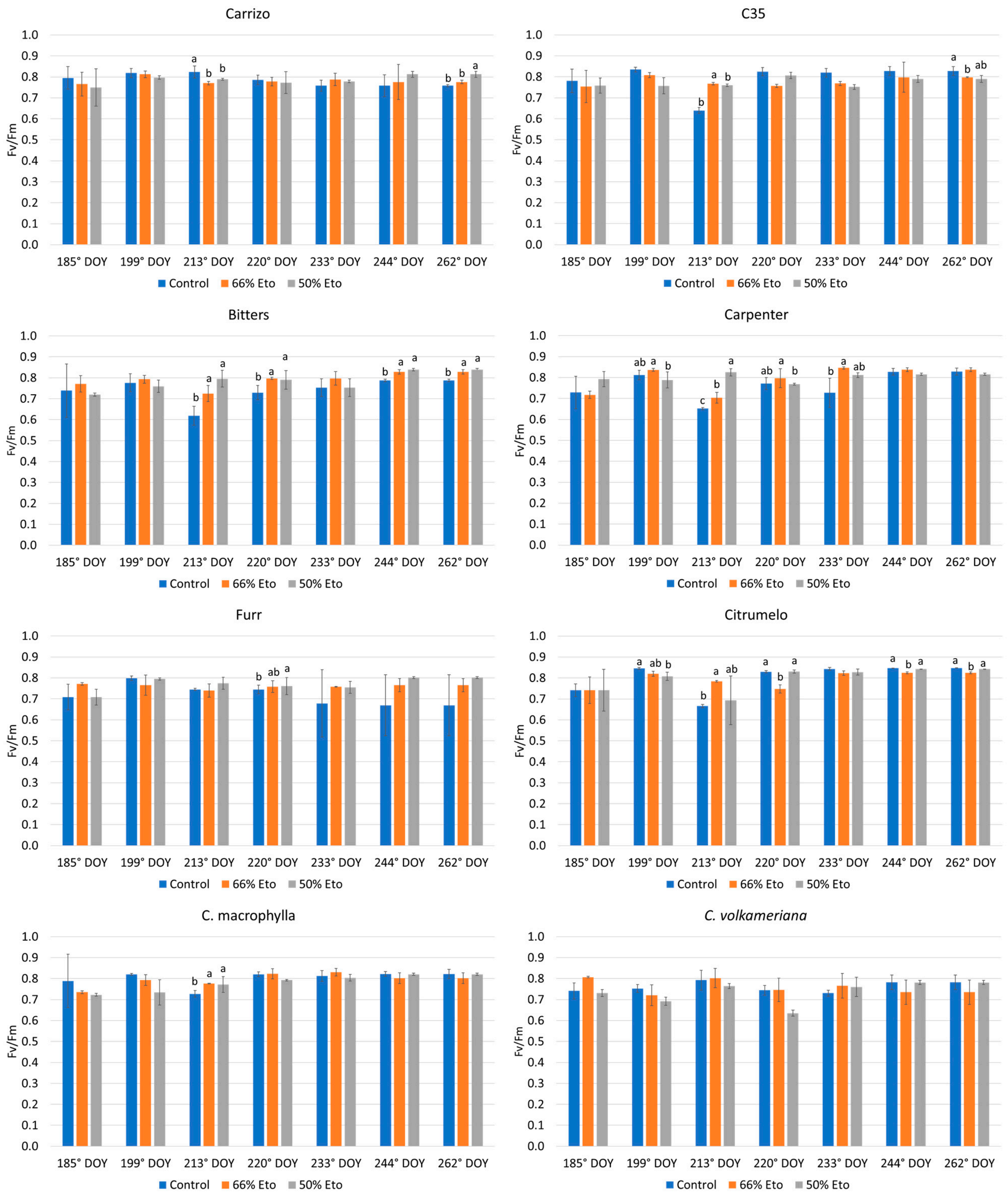

Figure S2. Chlorophyll fluorescence (Fv/Fm) determined in 8 rootstocks subjected by water stress from 185 to 262 day of the year (DOY). Means ± standard deviation of the three treatments analyzed in triplicate are reported. Values without letters have no significant differences according to Fisher's LSD procedure at 95 % confidence level.
